# Oral Health Behaviour and Social and Health Factors in University Students from 26 Low, Middle and High Income Countries

**DOI:** 10.3390/ijerph111212247

**Published:** 2014-11-26

**Authors:** Karl Peltzer, Supa Pengpid

**Affiliations:** 1 ASEAN Institute for Health Development, Mahidol University, Salaya, Phutthamonthon, Nakhonpathom 73170, Thailand; E-Mail: supaprom@yahoo.com; 2 Department of Psychology, University of Limpopo, Turfloop Campus, Sovenga 0727, South Africa; 3 HIV/AIDS/STIs/and TB (HAST), Human Sciences Research Council, Private Bag X41, Pretoria 0001, South Africa; 4 Department of Research & Innovation, University of Limpopo, Turfloop Campus, Sovenga 0727, South Africa

**Keywords:** ocial determinants, mental health, university students, 26 countries

## Abstract

Poor oral health is still a major burden for populations throughout the world, particularly in developing countries. The aim of this study was investigate oral health behaviour (tooth brushing and dental attendance) and associated factors in low, middle and high income countries. Using anonymous questionnaires, data were collected from 19,560 undergraduate university students (mean age 20.8, SD = 2.8) from 27 universities in 26 countries across Asia, Africa and the Americas. Results indicate that 67.2% of students reported to brush their teeth twice or more times a day, 28.8% about once a day and 4.0% never. Regarding dental check-up visit, 16.3% reported twice a year, 25.6% once a year, 33.9% rarely and 24.3% never. In a multivariate logistic regression analysis, being a male, coming from a wealthy or quite well off family background, living in low income or lower middle income, weak beliefs in the importance of regular tooth brushing, depression and PTSD symptoms, tobacco use and frequent gambling, low physical activity, and low daily meal and snacks frequency were associated with inadequate tooth brushing (<twice daily). Further, being a male, older age, coming from a not well off or poor family background, living in low income or lower middle income, weak beliefs in the importance of regular tooth brushing, PTSD symptoms, illicit drug use, low physical activity, and low daily snacks frequency, skipping breakfast and inadequate fruit and vegetables consumption were associated with less than one annual dental care visit. Oral health behaviour among the students was found to be low. Various risk factors identified can be used to guide interventions to improve oral health behaviour among university students.

## 1. Introduction

Healthy habits and good oral hygiene, including twice daily tooth brushing, are critical in preventing gum disease and maintaining good oral health [1]. The prevalence of less than twice tooth brushing per day among university students seems to be higher in low and middle income than in high income countries, e.g., 52.2% in India [2], 35% in Lebanon [3], 32% in Turkey [4]; 24% not regularly in Yemen [5]) than in high income countries (7.9% in Italy [6], 25% in USA [7]). Similarly, the prevalence of less than an annual dental check-up among university students also seem to be higher in developing countries (84% in Kenya [8], 92.2% in Nigeria [9], 70% in Turkey [4]; 42.7% in India never had a dental visit [2] and 48.2% in Iran never visited the dentist [10]) than in industrial countries (41% in Finland [11] and 40.1% in Italy [6]).

Various factors have been found to be associated with suboptimal tooth brushing among young people, including sociodemographics such as men [3,7,12,13,14,15,16] and lower socioeconomic status [17,18,19]; poor oral health attitude [2]; addictive risk behaviour such as smoking [5,13] and alcohol and cannabisuse [20]; lack of exercises [3], dietary behaviour, including infrequent fruits and/or vegetables consumption [21,22,23], frequent servings of chocolate, candy or chips/day [3], and poor mental health or psychological distress [21]. Less than annual or no dental visits has been found to be associated with being male [15,24,25,26,27], lower socioeconomic status [16,26,28], younger age [9]; poor oral health attitudes [25]; health risk behaviour, including infrequent tooth brushing [26], smoking [29] and anticipation of painful treatment [9].

Few studies have been conducted on oral health in university students, in particular in low and middle income counries. Therefore, the aim of this study was to investigate oral health behaviour (tooth brushing and dental attendance) and associated factors in low, middle and high income countries. The two hypotheses being that (1) male university students from lower socioeconomic backgrounds, having low physical activity, having more addictive risk behaviours, poorer dietary behaviour and poorer mental health are more likely to have poor tooth brushing behaviour, and (2) male university students from lower socioeconomic backgrounds and risk behaviours are more likely to have less than annual or no dental visits. This study adds to the existing research, in particular in the group of university students who are in a period of transition to take on personal responsibility for oral health behaviours and who will be future leaders and role models in their societies.

## 2. Methods

### 2.1. Sample and Procedure

This cross-sectional study was carried out with a network of collaborators in participating countries (see Acknowledgments). The anonymous, self-administered questionnaire used for data collection was developed in English, then translated and back-translated into languages (Arabic, Bahasa, French, Lao, Russian, Spanish, Thai, Turkish) of the participating countries. The study was initiated through personal academic contacts of the principal investigators. These collaborators arranged for data to be collected from intended 400 male and 400 female undergraduate university students aged 16–30 years by trained research assistants in 2013 in one or two universities in their respective countries. The 27 universities involved were located in the capital cities or other major cities in the participating countries. Research assistants working in the participating universities asked classes of undergraduate students to complete the questionnaire at the end of a teaching class. Classes were selected through a stratified random sample procedure. The students who completed the survey varied in the number of years for which they had attended the university. A variety of majors were involved, including education, humanities and arts, social sciences, business and law, science, engineering, manufacturing and construction, agriculture, health and welfare and services. Informed consent was obtained from participating students, and the study was conducted in 2013. Participation rates were in most countries over 90%. Ethics approvals were obtained from institutional review boards from all participating institutions.

### 2.2. Measures

#### 2.2.1. Oral Health Behaviour

Oral health behaviour was assessed with two questions, (1) “Do you brush your teeth? … Twice or more a day, about once a day, less than once a day, and seldom or never” [30] and (2) “How frequently do you go for dental checkups?...Twice a year, once a year, rarely and never” [12].

#### 2.2.2. Socio-Demographic

Socio-demographic questions included age, gender, and socioeconomic background were assessed by rating their family background as wealthy (within the highest 25% in “country”, in terms of wealth), quite well off (within the 50% to 75% range for their country), not very well off (within the 25% to 50% range from “country”), or quite poor (within the lowest 25% in their country, in terms of wealth) [30].

#### 2.2.3. Health Beliefs

Health beliefs in the importance of brushing teeth regularly were assessed with one question. The response option ranged from 1 = of very low importance to 10 = of very high importance [30].

#### 2.2.4. Mental Health

*Centres for Epidemiologic Studies Depression Scale (CES-D).* We assessed depressive symptoms using the 10-item version of the CES-D [31]. Scoring is classified from 0 to 9 as having a mild level of depressive symptoms, 10 to 14 as moderate depressive symptoms, and 15 representing severe depressive symptoms [32]. The Cronbach alpha reliability coefficient of this 10-item scale was 0.74 in this study.

*Post traumatic stress disorder (PTSD).* Breslau’s 7-item screener was used to identify PTSD symptoms in the past month [33]. Items asked included whether the respondent had experienced difficulties related to a traumatic experience (e.g., “Did you become jumpy or get easily startled by ordinary noises or movements?”). Consistent with epidemiological evidence, participants who answered affirmatively to at least four of the questions were considered to have a positive screen for PTSD [33]. The Cronbach alpha reliability coefficient of this 7-item scale was 0.75 in this study.

#### 2.2.5. Addictive Risk Behaviour

*Tobacco use* was assessed with the question: Do you currently use one or more of the following tobacco products (cigarettes, snuff, chewing tobacco, cigars, *etc.*)? Response options were “yes” or “no” [34].

*Binge drinking* was assessed with one item, “How often do you have (for men) five or more and (for women) four or more drinks on one occasion?” Response options ranged from 1 = never to 5 = daily or almost daily [35].

*Illicit drug use* was assessed with the question, “How often have you taken drugs in the past 12 months; other than prescribed by the health care provider.”

The *South Oaks Gambling Screen* (SOGS), a standardized measure of pathological gambling and gambling behaviours in their lifetime [36] was used to assess 9 different gambling behaviours, e.g., “Played cards for money.” Response options ranged from 1 = not at all to 3 = Once a week or more. Cronbach alpha for this 9 item scale was 0.91 in this sample.

#### 2.2.6. Physical Activity

*Physical activity* was assessed using the self-administered International Physical Activity Questionnaire (IPAQ) short version, for the last 7 days (IPAQ-S7S). We used the instructions given in the IPAQ manual for reliability and validity, which is detailed elsewhere [37]. We categorized physical activity (short form) according to the official IPAQ scoring protocol [38] as low, moderate and high.

#### 2.2.7. Dietary Behaviour Variables

Fruit and vegetable (FV) consumption was assessed with two questions ‘‘How many servings of fruit do you eat on a typical day?’’and ‘‘How many servings of vegetables do you eat on a typical day?’’ using the 24-h dietary recall data as the gold standard [39]. Insufficient FV consumption was defined as less than 5 servings of fruits and/or vegetables a day [39]. Additional dietary variables included: (a) frequency of having breakfast; (b) frequency of between-meal snacks and (c) number of meals a day [40].

### 2.3. Data Analysis

The data were analysed using STATA 11.00 (StatCorp LP, College Station, TX, USA). Descriptive statistics were used for reporting the proportion of oral health behaviour. We used univariate logistic regression, followed by multivariate unconditional logistic regression to obtain adjusted odds ratios (AOR) and associated 95% confidence intervals to assess the association between sociodemographic variables, health beliefs, addictive risk behaviour, low physical activity, poor mental health, dietary behaviour and oral health behaviour (<twice daily tooth brushing and less than annual dental attendance). All variables statistically significant at the *p* < 0.05 level in bivariate analyses were included in the multivariate model. *p* < 0.05 was considered significant. Variance inflation factor (VIF) and tolerance values for each model indicate multicollinearity was not a concern in any of the multivariate analyses. Since the study used a clustered design, country was included as a clustering variable in the regression models.

## 3. Results and Discussion

### 3.1. Sample Characteristics

In all, 21,639 university students were recruited, 20,222 agreed to participate and for 19,560 complete questionnaires of students in the age range of 16–30 years were available. The final sample included 19,560 university students (41.4% men and 58.6% women), with a mean age of 20.8 years (SD = 2.8). Overall, 67.2% reported to brush their teeth twice or more times a day, 28.8% about once a day and 4.0% never. Regarding dental check-up visit, 16.3% reported twice a year, 25.6% once a year, 33.9% rarely and 24.3% never. The overall prevalence of recommended tooth brushing frequency (twice or more a day) among university students differed by country, ranging from below 30% in Nigeria, India and Egypt, to about 90 or more percent in Thailand, Indonesia and Venezuela. Likewise, the dental check-up visit (at least once a year) differed by country, ranging from below 25% in Egypt, Ivory Coast, Nigeria, and South Africa to above 70% in Columbia, Venezuela, and Russia. The belief in the importance of tooth brushing was generally positively evaluated, with a mean of 8.7, on a scale from 1 to 10. However, university students from several countries had a weak belief in the importance of tooth brushing, e.g., Tunisia (5.7), Nigeria (6.5) and Egypt (7.2) (see Table 1).

### 3.2. Associations with Oral Health (Tooth Brushing and Dental Visit)

In multivariate logistic regression analysis, being a male, coming from a wealthy or quite well off family background, living in low income or lower middle income, weak beliefs in the importance of regular tooth brushing, poor mental health (depression and PTSD symptoms), addictive risk behaviour (current tobacco use and frequent gambling), low physical activity, and dietary behaviour (low daily meal and snack frequency) were associated with inadequate tooth brushing (<twice daily). Further, being a male, older age, coming from a not well off or poor family background, living in low income or lower middle income, weak beliefs in the importance of regular tooth brushing, poor mental health (PTSD symptoms), addictive risk behaviour (past year illicit drug use), low physical activity, and dietary behaviour (low daily snacks frequency, skipping breakfast and inadequate fruit and vegetables consumption) were associated with less than one annual dental care visit (see Table 2).

**Table 1:** Oral health behaviour prevalence by country.

Study Country	Tooth Brushing				Dental Check-up					Belief of the Importance of Tooth Brushing
		Twice or More per Day	About Once a Day	Less than Once a Day/Seldom or Never		Twice a Year	Once a year	Rarely	Never	
	N	%	%	%	N	%	%	%	%	M (SD)
All	19,560	67.2	28.8	4.0	19,399	16.3	25.6	33.9	24.3	8.7 (2.3)
Caribbean and South America										
Barbados ^4^	563	76.6	22.7	0.7	559	18.6	27.0	41.3	13.1	9.0 (1.5)
Grenada ^3^	430	85.1	14.2	0.7	431	21.8	32.9	34.1	11.1	9.2 (1.5
Jamaica ^3^	757	76.8	22.1	1.2	757	16.8	34.6	40.0	8.6	9.3 (1.5)
Colombia ^3^	816	94.2	5.8	0.0	816	42.0	37.6	18.4	2.0	9.4 (1.3)
Venezuela ^3^	554	84.7	14.1	1.3	552	34.1	40.6	22.8	2.5	9.3 (1.4)
Sub-Saharan Africa										
Cameroon ^2^	626	65.0	30.7	2.2	626	18.2	33.4	38.3	10.1	9.1 (1.8)
Ivory Coast ^2^	806	68.1	27.4	4.5	786	3.8	7.3	21.6	67.3	9.6 (1.9)
Madagascar ^1^	780	81.9	17.1	1.0	768	15.4	18.2	35.2	31.3	8.9 (2.0)
Mauritius ^3^	491	78.6	21.0	0.4	492	15.4	28.3	41.1	15.2	9.1 (1.7)
Namibia ^3^	494	59.7	39.5	0.8	486	11.3	21.8	24.9	42.0	9.3 (1.6)
Nigeria ^2^	755	20.9	70.5	8.6	731	3.3	8.9	28.5	59.4	6.5 (3.9)
South Africa ^3^	820	58.9	38.3	2.8	818	7.3	14.9	17.6	60.1	8.6 (2.4)
North Africa, Neareast and Central Asia										
Egypt ^2^	804	26.1	49.4	24.5	798	13.7	9.8	43.5	33.1	7.2 (3.0)
Tunisia ^3^	883	57.6	32.6	9.7	875	20.1	18.9	31.4	29.6	5.7 (3.4)
Turkey ^3^	795	52.6	37.4	10.1	768	16.9	28.4	39.2	15.5	8.4 (2.3)
Kyrgyzstan ^1^	836	64.1	33.5	2.4	836	18.9	32.5	38.0	10.5	9.0 (2.1)
Russia ^3^	784	71.7	25.4	2.9	883	29.5	42.0	24.9	3.6	8.9 (2.0)
South Asia and China										
Bangladesh ^1^	790	53.0	42.4	4.6	792	10.9	15.7	37.9	35.6	8.7 (2.3)
India ^2^	787	29.1	62.3	8.6	746	8.7	18.9	40.1	32.3	9.2 (2.0)
Pakistan	813	49.9	46.4	3.7	812	8.3	24.8	35.0	32.0	9.7 (0.7)
China ^3^	1102	79.3	19.6	1.1	1102	9.7	33.0	38.8	18.4	8.1 (1.7)
Southeast Asia										
Indonesia ^2^	749	89.9	9.2	0.9	742	16.3	15.5	45.8	22.4	9.5 (1.5)
Laos ^2^	806	77.0	21.1	1.9	806	11.9	20.3	30.9	36.8	8.8 (2.4)
Philippines ^2^	780	90.9	8.8	0.3	781	18.2	38.3	38.2	5.4	9.3 (1.3)
Singapore ^4^	885	77.6	20.8	1.6	887	17.6	31.1	38.7	12.6	8.2 (1.8)
Thailand ^3^	854	89.0	9.5	1.5	849	20.7	35.7	32.7	10.8	9.0 (1.7)

**^1^** Low income country; **^2^** Lower middle income country; **^3^** Upper middle income country; **^4^** High income country (Source: [41]).

**Table 2:** Logistic regression analyses predicting oral health (tooth brushing and dental visit).

Variables (N,% or M, SD)	Tooth Brushing < Twice Daily		Dental Visits < Yearly	
	Crude OR (95% CI)	Adjusted OR (95% CI)	Crude OR (95% CI)	Adjusted OR (95% CI)
Socio-Demographic Variables				
*Gender*				
Female (58.3%)	1.00	1.00	1.00	1.00
Male (41.7%)	2.06 (1.94–2.19) ***	2.12 (1.95–2.31) ***	1.50 (1.41–1.59) ***	1.53 (1.42–1.65) ***
*Age in years*				
16–19 (34.1%)	1.00		1.00	1.00
20–21 (35.5%)	1.06 (0.89–1.14)	---	1.13 (1.05–1.21) ***	1.06 (0.97–1.15)
22 or more (30.4%)	1.09 (0.99–1.16)		1.32 (1.22–1.42) ***	1.14 (1.04–1.25) **
*Wealth*				
Not well off/Poor (46.4%)	1.00	1.00	1.00	1.00
Wealthy/ Quite well off (53.6%)	1.50 (1.41–1.59) ***	1.43 (1.31–1.55) ***	0.70 (0.66–0.74) ***	0.66 (0.61–0.71) ***
*Country income*				
Upper middle income/High income (49.8%)	1.00	1.00	1.00	1.00
Low income/Lower middle income (50.2%)	2.12 (1.99–2.26) ***	1.65 (1.51–1.81) ***	2.22 (2.09–2.36) ***	2.15 (1.98–2.33) ***
**Health Beliefs**				
Weak beliefs in importance of regular tooth brushing (27.5%) (1–8 *vs.* 9–10) (base = 9–10)	2.24 (2.10–2.39) ***	1.85 (1.69–2.43) ***	1.34 (1.25–1.43) ***	1.34 (1.23–1.47) ***
**Poor Mental Health**				
Depression symptoms (≥15; severe) (12.8%) (base ≤ 15)	1.45 (1.34–1.58) ***	1.34 (1.19–1.51) ***	1.11 (1.02–1.21) *	1.03 (0.92–1.16)
PTSD symptoms (≥4) (20.9%) (base ≤ 4)	1.40 (1.30–1.51) ***	1.22 (1.10–1.35) ***	1.24 (1.15–1.34) ***	1.19 (1.08–1.31) ***
**Addictive Risk Behaviour**				
Current tobacco use (12.1%) (base ≤ 4 weeks tobacco use)	1.53 (1.41–1.67) ***	1.17 (1.04–1.32) ***	0.99 (0.91–1.08)	---
Binge drinking (past month) (11.8%) (base ≤ past months)	0.78 (0.71–0.86) ***	0.74 (0.64–0.84) ***	0.64 (0.59–0.79) ***	0.67 (0.60–0.75) ***
Past year illicit drug use (17.4%) (base ≤ past year)	0.95 (0.89–1.03)	---	1.40 (1.30–1.50) ***	1.23 (1.13–1.35) ***
Gambling (>once a week) (8.2%) (base ≤ 1 week)	1.30 (1.17–1.44) ***	1.23 (1.06–1.43) ***	1.02 (0.92–1.13)	---
**Low Physical Activity** (47.5%) (base = moderate or high physical activity)	1.23 (1.16–1.30) ***	1.15 (1.06–1.25) ***	1.17 (1.10–1.24) ***	1.09 (1.01–1.18) *
**Dietary Behaviour**				
Daily meal frequency [2.6 (0.7] (continuous variable)	0.77 (0.73–0.80) ***	0.87 (0.82–0.93) ***	0.81 (0.78–0.85) ***	0.96 (0.91–1.01)
Daily snacks frequency [1.5 (0.9)] (continuous variable)	0.91 (0.88–0.94) ***	0.92 (0.88–0.97) ***	0.86 (0.83–0.89) ***	0.88 (0.85–0.92) ***
Skips breakfast (46.2%) (base = not skip breakfast)	1.24 (1.17–1.32) ***	1.07 (0.98–1.17)	1.25 (1.18–1.33) ***	1.09 (1.01–1.18) *
Fruit and Vegetables (<5 servings/day) (82.8%) (base ≥ 5 servings/day)	1.15 (1.05–1.25) **	1.05 (0.94–1.18)	1.15 (1.06–1.24) ***	1.12 (1.01–1.24) *

*** *p* < 0.001; ** *p* < 0.01; * *p* < 0.05.

### 3.3. Discussion

The study found, among a large sample of university students from 26 low, middle and high income countries across Asia, Africa and the Americas, a large group of 32.8% engaged in inadequate or no tooth brushing, and 58.2% rarely or never had gone for a dental check-up visit. These findings seem to confirm low rates of tooth brushing and dental attendance in developing economy countries [2,3,4,5,8,9,10] compared to high income countries [6,7,11].

However, the study found a large country variation in the overall prevalence of inadequate tooth brushing and less than annual dental visits, which in part confirms findings from previous studies of poor oral health practices in India, Nigeria, South Africa, and Turkey [2,4,9,42], and on the other hand countries with more optimal oral health practices such as Colombia [14] and Thailand [43]. Studies among dental students in different cultures showed that oral health behaviour can largely differ between countries, which could be explained by different cultural and health education systems [4,44,45]. For example, in some cultures the toothpick is commonly used as a dental cleaning device or different perceptions on putting off going to the dentist until having toothache [4,44]. It is further possible that in our study countries with poor oral health practices (India, Nigeria, South Africa, Turkey) oral hygiene education was not sufficiently integrated into the secondary school system.

In concordance with a number of other studies [3,7,12,13,14,15,16,24,25,26,27], the study found across the countries that men engaged more frequently in poor oral health behaviour (less frequent tooth brushing, less or never dental attendance). It is possible that women care more for body and appearance, and thus their oral health than men [2,3]. Lack of dental attendance was in agreement with another study [9] associated with younger age.

Further, the study found that living in a low income or lower middle income was associated with poor oral health behaviour (tooth brushing and dental attendance), the same was true regarding coming from a not well off or poor family background and poor dental attendance, while coming from a wealthy or quite well off family background was associated with poor tooth brushing behaviour. Previous studies have generally found that lower socioeconomic background was associated with a poorer oral health behaviour (tooth brushing and dental attendance) [16,17,18,19,26,28]. It is possible that university students with lower socioeconomic status were less likely to go for regular dental check-ups because they were not able to pay for the dental services.

Unexpectedly, students from more affluent economic backgrounds were less likely to brush their teeth twice of more a day. It is possible that in this group other factors such as lack of knowledge and cultural values about the importance of tooth brushing impact on poor oral health behaviour [26]. University students from lower economic backgrounds may be equally exposed to oral health information and communication than students from higher economic backgrounds, and engage in oral health behaviour (tooth brushing).

After adjusting for relevant socio-demographic variables, the current study findings indicate associations between poor oral health behaviour and weak beliefs in the importance of regular tooth brushing, poor mental health, and health risk behaviours, including addictive risk behaviours, low physical activity, inadequate dietary behaviours (skipping breakfast and insufficient fruit and vegetable consumption), which is largely consistent with findings of previous research [2,3,5,13,20,21,22,23,25,29]. Contrary to some previous findings [3,20], this study found that binge drinking and more frequent daily meals and snacks, was found to be protective of poor oral health behaviour. For some of these findings the reports in the literature has been conflicting and might also come from different measurement instruments [3]. The significant relationship of oral health behaviour with the attitudes of the students, indicates the importance of attitude and beliefs in influencing the behaviour, which should be utilized in planning preventive oral health promotion programmes. Present results seem to generally indicate an overlap between oral and general health behaviour, which could imply that oral and general health behaviour should be approached jointly in health promotion lifestyle programmes [46].

#### Study Limitations

This study had several limitations. The study was cross-sectional, so causal conclusions cannot be drawn. The investigation was carried out with students from one or two universities in each country, and inclusion of other centres could have resulted in different results. University students are not representative of young adults in general, and the oral health behaviour levels, sociodemographic and health risk behaviour variables may be different in other sectors of the population. A further limitation of the study was that all information collected in the study was based on self-reporting. It is possible that rates of oral health behaviours were under or over reported. However, a previous study found that the self-reported dental care use was valid for some service types [47]. Further, the assessment of oral health behaviour was limited to tooth brushing and dental attendance, and other oral hygiene knowledge and behaviours such as knowledge of fluoridated toothpastes, frequency of brushing with fluoride toothpaste, frequency of toothbrush renewal, use of dental floss, and ‘sugar behaviour’ [2,48] and oral health status [49,50] should be assessed in future studies. Finally, no data of variety of social factors in the same country, and no link between the two poor oral health behaviours and oral diseases (periodontal diseases) were assessed and include an additional limitation of the study

## 4. Conclusions

This study confirms low rates of tooth brushing and dental attendance among university students in different cultures across Africa, Asia and the Americas. Various factors identified, such as socioeconomic status, weak beliefs in the importance of regular tooth brushing and co-occurrence of general health risk behaviour can be used to guide interventions to improve oral health behaviour among university students. Oral health promotion programmes targeting the young adult population could help to improve their attitude and oral health behaviour to achieve good oral health.
